# Generalized anxiety disorder among diabetic patients visiting gharyan-polyclinic in Libya during COVID-19 pandemic

**DOI:** 10.1192/bjo.2021.705

**Published:** 2021-06-18

**Authors:** Ahmed Khrwat, Abduraoof Saadawi

**Affiliations:** 1 faculty of medicine, University of Gharyan; 2 Gharyan polyclinic

## Abstract

**Aims:**

To estimate the prevelance of Generalized anxiety disorder (GAD) in adult patients with diabetes mellitus (T1DM or T2DM) during COVID-19 pandemic.

**Method:**

Random sample of 115 Adult Libyan patients (≥18 years) were drawn from 1200 Medical records of diabetic patients previously diagnosed in a primary care clinic (Gharyan polyclinic,South of Tripoli,West of Libya). Patients were recruited and diagnostically interviewed through outpatient visits and through Phone calls. Anxiety was assessed using Generalized Anxiety Disorder 7-item instrument (GAD-7), personal information, Co morbidities and History of COVID-19 infection within period of 3 weeks.

**Result:**

The statistical analysis done by SPSS version 23, using ANOVA test. The GAD-7 scores ranged from 0 to 19 for the diabetic patient, 82 patients scores ranged from 0 to 4 with varying degrees of non-signifacant to subsyndromal symptopms of Generalized anxiety disorder, 24 patient with Mild GAD, 7 patients with moderate GAD and 2 patients with severe GAD. (P value = 0.000)

**Conclusion:**

GAD is present in 28% of the patients who participated in the study. Additional epidemiological studies are needed to determine the prevalence of anxiety in the broader population of persons with diabetes.

